# Uterine Artery Pseudoaneurysm in the Setting of Delayed Postpartum Hemorrhage: Successful Treatment with Emergency Arterial Embolization

**DOI:** 10.1155/2011/373482

**Published:** 2011-09-22

**Authors:** Ankur M. Sharma, Brent E. Burbridge

**Affiliations:** ^1^College of Medicine, University of Saskatchewan, Saskatoon, SK, Canada; ^2^Department of Medical Imaging, College of Medicine, University of Saskatchewan, Saskatoon, SK, Canada; ^3^Medical Imaging, Royal University Hospital, 103 Hospital Drive, Saskatoon, SK, Canada, S7N 0W8

## Abstract

Postpartum hemorrhage is a major cause of maternal mortality. Though uncommon, uterine artery pseudoaneurysm can follow uterine dilatation and curettage (D + C) and needs to be considered in the differential diagnosis. This 30-year-old G1P1 woman presented with right upper quadrant pain and vaginal bleeding. She was afebrile but her white blood count was significantly increased (22.2 × 10^9^ /L). One week prior, she had undergone a Cesarean delivery which was complicated by hemolysis, elevated liver enzymes, and low platelet count syndrome (HELLP), fetal dystocia, and chorioamnionitis. Uterine dilatation and curettage (D & C) and placement of a Bakri intrauterine balloon, performed for suspected retained products of conception, failed to control her postpartum bleeding. The patient wished to have a hysterectomy only as a last resort in order to preserve fertility. Emergency uterine artery angiography revealed a left uterine artery pseudoaneurysm and contrast extravasation. The patient was successfully treated with selective embolization. Computed tomography (CT) later revealed dehiscence of her uterine Cesarean section incision with an intra-abdominal fluid collection. This collection was drained. She also developed disseminated intravascular coagulopathy (DIC) syndrome as well as multiple pulmonary emboli which were both successfully treated. We discuss this unique case of uterine artery pseudoaneurysm with associated uterine dehiscence.

## 1. Introduction

Postpartum hemorrhage can occur within 24 hours or up to 6 weeks after delivery [1]. It is commonly caused by endometritis or retained products of conception, but other less common causes such as choriocarcinoma, uterine arteriovenous malformation, and uterine artery pseudoaneurysm should not be overlooked, as they can cause significant morbidity and mortality [2–4].

## 2. Case Report

A 30-year-old G1P1 women presented to the Emergency Department (ED) with right upper quadrant pain, dizziness, and mild vaginal bleeding. One week prior she had undergone a Cesarean section (C-section) due to dystocia during which the fetus died, probably secondary to sepsis. The pregnancy was complicated by chorioamnionitis and hemolysis, elevated liver enzymes, and low platelet count, that is, HELLP syndrome. 

Vital signs revealed tachycardia (heart rate—116) and a blood pressure of 131/87 mm Hg. Physical exam revealed diffuse abdominal tenderness and mild abdominal distention. 

Laboratory tests demonstrated an elevated white count of 22.2 × 10^9^ /L, a hemoglobin of 104 g/L and a platelet count of 406 × 10^9^ /L. Liver function tests, coagulation factors, and other chemistry tests were normal. The patient was admitted with suspected systemic infection and for the management of her bleeding per vagina. 

Transabdominal and transvaginal pelvic ultrasound examinations acquired while in the ED showed hypoechoic material within a distended endometrial cavity measuring 2 cm in diameter, along with an anterior uterine wall irregularity related to the recent C-section and a small amount of free pelvic fluid. Doppler ultrasonography of the uterus and adnexal regions was negative. Abdominal ultrasound revealed subcutaneous edema and a left pleural effusion. There was also a small amount of fluid beneath the cutaneous C-section wound associated with a 1.0 × 1.5 cm midline gap in the rectus muscles.

On the same day, as her admission, the patient was given intravenous fluids and initially treated with Misoprostol (Pfizer Canada, Kirkland, QC, Canada), to treat what felt to be retained products of conception, but the vaginal bleeding continued and she was taken to the operating room (OR) where a dilatation and curettage (D & C) of the uterus was performed. The D & C failed to stop the uterine bleeding, and it actually became more brisk resulting in the subsequent placement of a Bakri intra-uterine balloon (Cook Canada Inc., Stouffville, ON, Canada). While transferring the patient postoperatively from the surgical table to an ICU bed, profuse vaginal bleeding continued unabated, and the decision was made to call interventional radiology.

By this time, the patient had received 8 units of packed red blood cells, 4 units of fresh frozen plasma, 5 units of platelets, and 1 liter of IV fluid for resuscitation.

**Figure 1:**
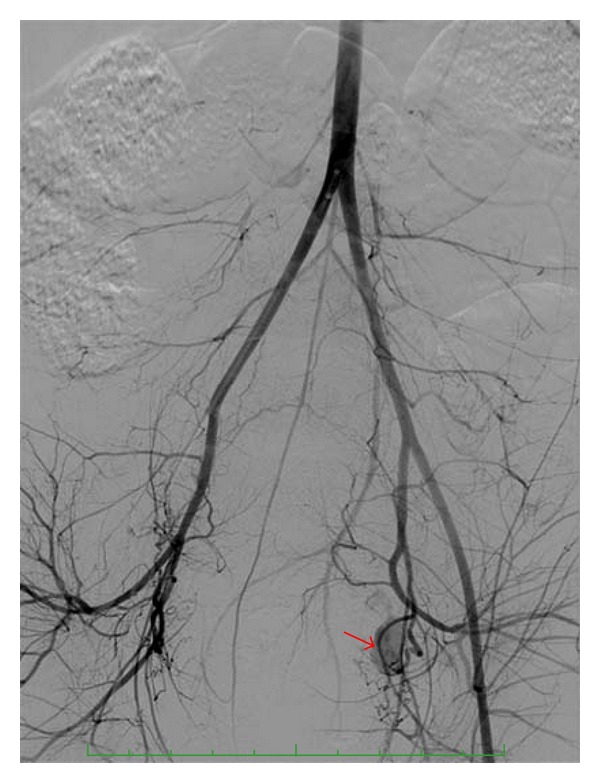
The subtracted aortogram image demonstrates generalized arterial spasm, spasm of the right common femoral artery, and a left uterine artery pseudoaneurysm (arrow).

The patient was immediately transferred to the angiography suite directly from the OR. An endotracheal tube was in situ, and the patient was under general anesthesia. She was being aggressively resuscitated with intravenous fluids and blood products. Upon attendance to the angiography suite, it was found that the patient's femoral artery was not palpable. Femoral artery access was performed with ultrasound guidance. An abdominal aortogram demonstrated a generalized diminution in arterial caliber felt to be associated with systemic hypotension. In addition, extravasation of contrast was seen from the left uterine artery during the aortogram (Figure 1).

**Figure 2:**
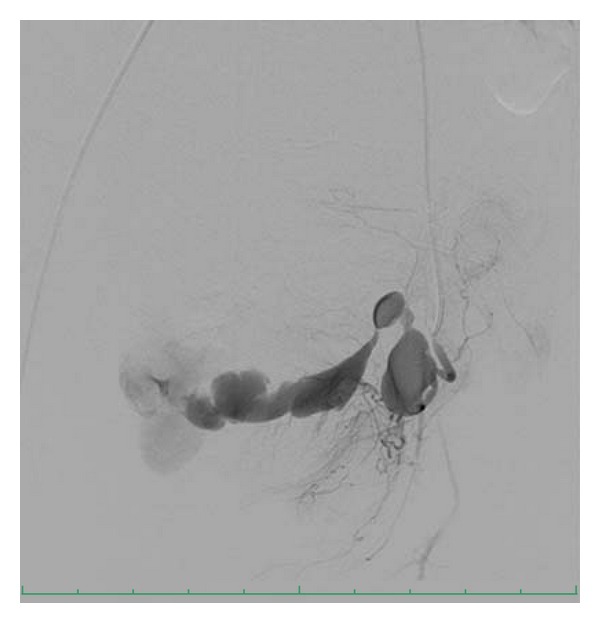
A selective left uterine artery angiogram reveals a pseudoaneurysm with prompt and florid extravasation of contrast into the pelvis.

Selective left uterine artery catheterization revealed an irregular collection of contrast agent consistent with a pseudoaneurysm (Figure 2). It was also apparent that there was frank extravasation of contrast agent from the pseudoaneurysm into an unknown space adjacent to the uterus. Extravasated contrast did not enter the vagina during angiography.

**Figure 3:**
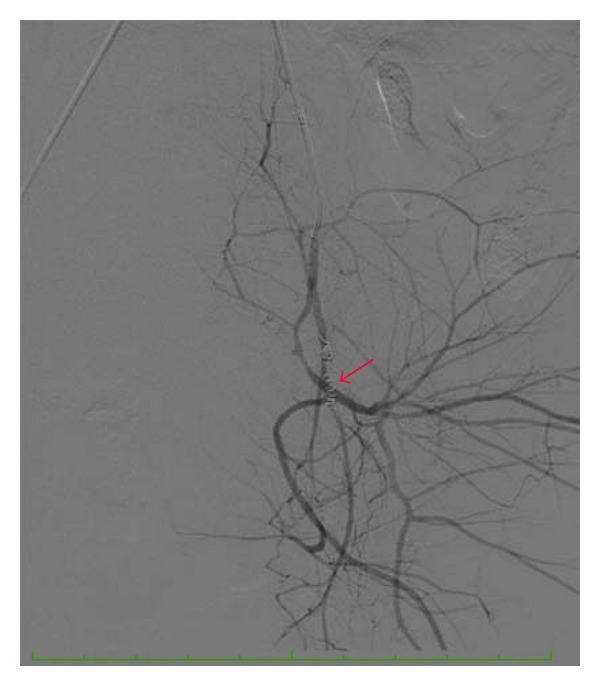
Postembolization angiography of the anterior division of the left internal iliac artery illustrates the embolization coils (arrow). There is no evidence of a pseudoaneurysm or of contrast extravasation.

Subsequently, the left uterine artery was catheterized with a 5F Roberts uterine artery catheter (Cook Canada Inc., Stouffville, ON, Canada). The left uterine artery was embolized using 4-4 cm long, 5 mm tapering to 3 mm, 0.35 inch diameter, Tornado, intra-arterial metallic coils (Cook Canada Inc., Stouffville, ON, Canada). Bleeding per vagina subsided significantly. No other source of contrast extravasation was identified at angiography in either hemi-pelvis, particularly on the left side (Figure 3). The patient was transferred to the Intensive Care Unit for further care.

Shortly after the embolization procedure, the patient developed DIC syndrome (Hemoglobin 69 g/L, Platelets 79 × 10^9^ /L, INR 1.5, APTT—65 seconds). Her blood pressure was 80/40 mm Hg. She was successfully treated with platelets, fresh frozen plasma, and packed red cells and was stabilized. One day after ICU admission, her blood pressure had stabilized and the bleeding per vagina had ceased.

**Figure 4:**
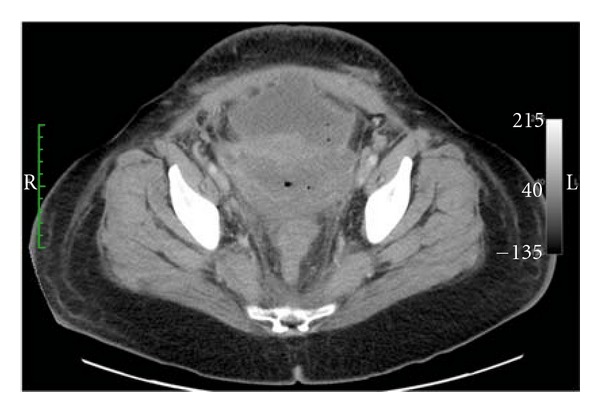
This axial CT-obtained cranial to the urinary bladder reveals disorganized lower uterine wall anatomy, a peritoneal fluid collection, and scattered gas bubbles in the uterus and peritoneum.

**Figure 5:**
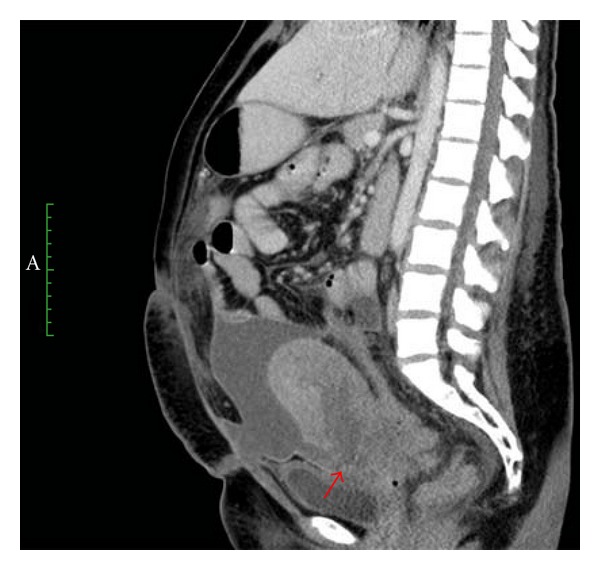
This sagittal CT of the abdomen better demonstrates the dehiscence of the anterior, lower uterus (arrow), and the peritoneal fluid. The endometrial cavity is distended with fluid that is contiguous with the peritoneal space.

Three days after the uterine artery embolization, the patient developed diarrhea and a fever of 38.9°C, as well as, a cough producing greenish-yellow sputum. CT of the abdomen and pelvis, in the axial plane, demonstrated intra-abdominal fluid and disorganized anatomy in the lower anterior wall of the uterus associated with scattered gas bubbles in the uterus and peritoneal fluid (Figure 4). In the sagittal plane, it was evident that there was an anterior uterine incision dehiscence with peritoneal fluid contiguous with the endometrial cavity (Figure 5).

The intra-abdominal fluid collection was successfully drained by Interventional Radiology and the patient's fever and diarrhea resolved with drainage and antibiotic treatment. 

Five days after abscess drainage, the patient complained of shortness of breath, chest pain, and back pain. A CT pulmonary embolism study revealed findings consistent with subsegmental pulmonary emboli in the left lower lobe. She also had airspace opacification in both lower lobes and the right middle lobe, compatible with bilateral bronchopneumonia, and moderate bilateral pleural effusions. Treatment with intravenous anticoagulation was followed by conversion to oral anticoagulation without any bleeding complications. The patient did well on this treatment regimen.

Subsequently, the patient was discharged from hospital 22 days after she was first seen in the Emergency Department.

## 3. Discussion

The cause of the arterial injury, resulting in the formation of the uterine artery pseudoaneurysm in this patient, is uncertain. It could have been due to the original uterine incision for the Cesarean section, the delivery of the fetus at Cesarean, or the suturing of the Cesarean-section incision. The catastrophic bleeding encountered in this instance did not begin until after the uterine dilatation and curettage. It is feasible to suspect that the dilatation and curettage had the net result of perforating the uterus leading to a dehiscence of the uterine Cesarean incision and creating a pseudoaneurysm. The extravasated contrast seen on the selective uterine artery angiograms was most likely entering the peritoneal cavity via the dehiscence.

Diagnosis of a pseudoaneurysm can be made by ultrasound, CT, or angiography. Initial treatment should include volume replacement and blood transfusions, as well as vaginal or uterine packing. If bleeding persists, other options include hysterectomy, surgical uterine artery ligation, or selective uterine artery embolization. 

First described in 1979, selective uterine artery embolization has evolved to offer such advantages as potentially preserving fertility, being minimally invasive, and having success rates of more than 90% [5–7].

## 4. Conclusion

In this case, the patient's uterine artery was successfully embolized preserving her uterus and terminating her life-threatening vaginal bleeding. There have been other case reports of successful uterine artery embolization in situations such as this, but none to our knowledge demonstrating uterine dehiscence associated with a uterine artery pseudoaneurysm [1–4].
